# The Microphthalmia-Associated Transcription Factor (MITF) and Its Role in the Structure and Function of the Eye

**DOI:** 10.3390/genes15101258

**Published:** 2024-09-27

**Authors:** Andrea García-Llorca, Thor Eysteinsson

**Affiliations:** 1 Department of Physiology, Biomedical Center, Faculty of Medicine, University of Iceland, 101 Reykjavík, Iceland; thoreys@hi.is; 2 Department of Ophthalmology, Biomedical Center, Faculty of Medicine, University of Iceland, 101 Reykjavík, Iceland

**Keywords:** *Mitf*, transcription factor, eye, retinal pigment epithelium, retina, mouse models, microphthalmia, retinal degeneration

## Abstract

Background/Objectives: The microphthalmia-associated transcription factor (*Mitf*) has been found to play an important role in eye development, structure, and function. The *Mitf* gene is responsible for controlling cellular processes in a range of cell types, contributing to multiple eye development processes. In this review, we survey what is now known about the impact of *Mitf* on eye structure and function in retinal disorders. Several mutations in the human and mouse *Mitf* gene are now known, and the effects of these on eye phenotype are addressed. We discuss the importance of *Mitf* in regulating ion transport across the retinal pigment epithelium (RPE) and the vasculature of the eye. Methods: The literature was searched using the PubMed, Scopus, and Google Scholar databases. Fundus and Optical Coherence Tomography (OCT) images from mice were obtained with a Micron IV rodent imaging system. Results: Defects in neural-crest-derived melanocytes resulting from any *Mitf* mutations lead to hypopigmentation in the eye, coat, and inner functioning of the animals. While many *Mitf* mutations target RPE cells in the eye, fewer impact osteoclasts at the same time. Some of the mutations in mice lead to microphthalmia, and ultimately vision loss, while other mice show a normal eye size; however, the latter, in some cases, show hypopigmentation in the fundus and the choroid is depigmented and thickened, and in rare cases *Mitf* mutations lead to progressive retinal degeneration. Conclusions: The *Mitf* gene has an impact on the structure and function of the retina and its vasculature, the RPE, and the choroid in the adult eye.

## 1. Introduction

The development of the eye and its structure and function in adulthood are dependent on a complex array of genes that includes several transcription factors. One of these transcription factors, which has been known since the middle of the last century to play a role in eye development, is the microphthalmia-associated transcription factor (MITF; OMIM: 156845) [1]. Over 80 years ago, Paula Hertwig, a German scientist, first observed mice with abnormally tiny eyes in the offspring of a male mouse that had been exposed to radiation, marking the beginning of microphthalmia-associated transcription factor (MITF) research. She correlated the observable characteristics to a new genetic mutation in a region she initially labeled as m [1], but later changed to *mi* [2]. Mutant homozygotes exhibited tiny, abnormal eyes and complete whiteness, and subsequent analysis revealed that they suffered from hearing impairments, with both of these characteristics being a result of the deficiency of melanocytes derived from the neural crest [3]. The *Mitf* gene belongs to the Myc supergene family of basic–helix–loop–helix–leucine (bHLHZip) transcription factors that contain TFE, TFE3, TFEB, and TFEC. The common feature among proteins in this family is the presence of a basic domain for DNA binding, as well as HLH and Zip domains for forming homo- or heterodimers [4,5]. In vertebrates, these genes are expressed in many cell types, with *Mitf* being notably abundant in neural-crest-derived melanocytes and in the retinal pigmen epithelium (RPE) [6,7,8]. Moreover, *Mitf* has a relatively high expression in mast cells, osteoclasts, kidney cells, and heart muscle cells, with lower expression in various other cell types [9,10,11,12]. As mentioned above, the RPE shows strong *Mitf* expression and if mutations occur, then that causes the RPE to become hyperproliferative, leading to a lack of pigmentation and affecting the neuroretina in the dorsal region. The regular retina keeps growing as usual in a tiny eye, leading to the formation of numerous folds and the eventual degeneration of the retina. The result is a complex colobomatous microphthalmia that greatly impairs image formation by the eye [8,13,14,15].

*Mitf* mutations impact melanocytes to different extent, with some mutations also affecting RPE cells, leading to eyes that are smaller than usual and lacking pigmentation, with retinal degeneration, ultimately leading to blindness [16,17]. Approximately half of the *Mitf* mutations are recessive, resulting in a phenotype only seen in homozygous animals. The other half are semidominant, causing white spotting and/or coat color dilution in heterozygotes [7,18,19,20]. *MITF* mutations in humans have been linked to Waardenburg syndrome (WS), a genetic condition causing both reduced skin pigmentation and hearing loss [21]. Some individuals with Tietz syndrome (OMIM: 103500), a genetic disorder marked by severe deafness and an overall lack of pigmentation, have also been shown to carry *MITF* mutations. Because of the significant similarities in physical characteristics, Tietz syndrome is viewed as a more severe form of WS2A (OMIM: 193510). Both conditions are dominant, and all documented instances involve patients with heterozygous genotypes [22,23]. Additionally, compound heterozygous genotypes were found in individuals suffering from a novel syndrome called coloboma, osteopetrosis, microphthalmia, macrocephaly, albinism, and deafness (COMMAD; OMIM: 617306) [24]. The mutation was identified separately by two research teams using either whole-genome sequencing or candidate gene methods, and it alters a sumoylation sire that was previously investigated in vitro [25,26,27]. More than 40 mutations in the human and mouse *Mitf* genes are known, and some of their effects on structure and function in the eye of mice have been examined, while there are very limited data available on the effects of such mutations in human eyes. In this review, we will discuss the effect of the known *Mitf* mutations on eye structure and function, the importance of *Mitf* in eye development, and the role of this gene in the RPE. Finally, retinal disorders associated with *Mitf* will also be discussed.

## 2. The Role of *Mitf* in the RPE

There is evidence that the RPE is involved in a variety of cellular functions [28,29], but research on these functions has been hampered by the fact that RPE cells are difficult to work with, and in addition they are not the only pigmented cells in the eye that are involved in retinal physiology and pathology. In the iris and the ciliary body, there are many neural-crest-derived pigmented cells, and such cells are also present in abundance in the choroid [30]. However, *Mitf* does not act in isolation in the eye; instead, it acts in conjunction with other transcription factors such as *Pax*, *Otx*, and *Vsx*, some of which play a role in eye development and RPE specification. In addition, MITF has, either through the direct transcriptional control of target genes or through other means, an effect on the expression of proteins in RPE cells [11] involved in processes like pigmentation, ion transport across the RPE, oxidative stress, the phagocytosis of photoreceptor outer segments, and the autophagy of cellular debris. Of these processes, perhaps the involvement of MITF in ion transport across the RPE is the least examined, but the expression of some of the ion channels on RPE cells is affected by MITF.

### Mitf and Ion Transport across the RPE

In this review, we chose to address the putative role of MITF in regulating ion transport across the RPE, since this has been addressed only to a limited extent. However, the other functions of the RPE in relation to regulation by MITF are numerous and have been reviewed in detail elsewhere [11].

One of the target genes of *Mitf* is the transient receptor potential cation channel subfamily M member 1 (*TRPM1*) gene [31]. It is primarily expressed on pigmented cells in the eye and skin [32,33], but is also expressed on retinal neurons [34]. The TRPM1 ion channel, like most TRP channels, is a non-selective cation channel with good permeability for Ca^2+^ ions [34]. The presence of the TRPM1 channel has been demonstrated in both mouse [35] and human RPE cells [36], and it has been shown to mediate calcium flux across the cell membranes [35]. It has been shown that the activation of TRPM1 on RPE cells triggers lysosomal calcium efflux, and thus increased levels of calcium in the cytoplasm [36], but this is not due to extracellular influx, so it is not clear whether TRPM1 channels are involved in ion transport across the RPE. It has been found that *MITF* knockdown using MITF siRNA in cultured human fetal RPE (hfRPE) cells decreases the expression of the *TRPM1* gene in these cells [37]. The knockdown of MITF in hfRPE cells cultured on transwells leads to a reduction in transepithelial resistance (TER) across the transwell, indicating a loss of RPE barrier function, but this could be reversed by the transfection of pre-miR-204/211 (micro RNA 211 resides in the sixth intron of TRPM1 and is thus regulated by MITF) [37]. However, these findings do not establish that TRPM1 on RPE cells is involved in ion transport, although this cannot be ruled out, since the changes in TER observed with a change in the expression of MITF and miR-204/211 are due to changes in the expression of tight junction proteins like ZO-1 and MCT3 [37]. However, it has been found recently that the conditional, RPE-specific deletion of mouse miR-204/211 leads to slow retinal degeneration with dysfunction and loss of photoreceptors, together with retinal inflammation [38], indicating that TRPM1 channels in the RPE play a role in maintaining the viability of photoreceptors. TRPM1 is also expressed in the mouse neuroretina, in photoreceptors, and in the outer nuclear and plexiform layers [39], although it is not clear whether *MITF* regulates its transcription there as it does in the RPE.

The electroretinogram (ERG) method is used to record the electrical response of the retina to light stimuli using corneal electrodes, and is a standard method used to detect retinal dysfunction in both patients and animal models. The ON-bipolar cells of the retina show high TRPM1 protein levels [40]. TRPM1^−/−^ mutant mice have negative ERG responses, with no positive b-waves generated by retinal bipolar cells [40,41], and their ON-bipolar cells do not show “light responses” in retinal slices simulated by blocking the metabolic glutamate 6 (mGluR6) receptor, presumably due to the absence of TRPM1 [40]. Deletion of the mGluR6 receptor renders the TRPM1 channel in the retina inactive [42]. The exact role of TRPM1 in ON-bipolar cells interacting with other membrane proteins, like nictalopin and glutamate receptors, is complex [43,44], but it is now known that mutations in the human *TRPM1* gene lead to autosomal recessive complete congenital stationary night blindness (CSNB; OMIM: 613216) [45]. All this indicates that the TRPM1 plays a fundamental role in mediating the rod ON-bipolar cell transmission of the light response to the inner retinas of both humans and mice, but since negative ERG responses have not been seen in any mutant mice with *Mitf* mutations it is unlikely that *Mitf* plays a role in regulating TRPM1 function in the neuroretina, and it likely only impacts the RPE at the back of the eye.

Bestrophin-1 is an ion channel that is expressed in the RPE and encoded by the *BEST1/VMD2* gene (now referred to as *BEST1*), which has been shown to be an *MITF* target gene [46], in addition to *OTX2* [47] and *SOX9* [48]. It is primarily expressed on the basolateral side of the RPE [49]. It has been shown that *MITF* activates the *BEST1*-promoter through an E-box sequence that is located -42 bp upstream of the initiation site, thus controlling *BEST1* expression [46]. *MITF* and the homeobox protein *OTX2*, another transcription factor in the RPE, are co-localized in the nuclei of RPE cells [50]. Unlike *MITF*, *OTX2* is also expressed in the neuroretina, but in the RPE, *OTX2* modulates the expression of *BEST1* by increasing the promoter activity via binding to two separate OTX-binding sites on *BEST1* [47]. An HMG-box transcription factor from the SRY (*SOX*) family, *SOX9*, is strongly expressed in the mouse RPE [51]. The *SOX* family of transcription factors has a high-mobility group domain that is a DNA-binding motif, which is like the mobility group domain of sex-determining region Y (SRY). The *SOX9* gene expressed in RPE cells has been found to bind to the −154 to −104 bp segment of *BEST1,* which contains regulatory elements, and plays a key role in the regulation of *BEST1* in the RPE. In addition, *SOX9* in the RPE interacts physically with *MITF* and *OTX2*, and with these transcription factors regulates the synergistic activation of the *BEST1* promoter [48]. Thus, *MITF,* in concert with other transcription factors, regulates the expression of the Bestrophin-1 ion channel, primarily on the basolateral side of the RPE, which suggests a role for *MITF* in regulating ion transport across the epithelial tissue. However, the actual function of the bestrophin-1 channel in the RPE and its role in RPE physiology are still somewhat unclear. It is known that the bestrophin-1 channel is both a Ca^2+^-activated chloride channel (CaCC) [52] and functions as a regulator of intracellular calcium signaling by altering the kinetics and voltage dependence of voltage-gated calcium channels (VDCC) [53]. Mutations in the *BEST1* gene have been related to five distinct retinal degenerative diseases [54], and one unresolved issue is which function of the channel relates to each of these. This group of diseases is referred to as bestrophinopathies, and the most common of these is Best’s disease (Best vitelliform macular dystrophy; OMIM: 611809). Best’s disease manifests itself in the posterior pole of the eye, with the development of yellow, vitelliform lesions that are elevated in the macula. The form of the lesion then changes in stages, with a worsening of the central vision. There is evidence indicating that *BEST1* mutations alters the regulation of VDCC kinetics via bestrophin-1 [55,56], and this reduces the response to light in electrooculogram (EOG) in patients with Best’s disease. In this test, the standing potential across the eyes of the patient is measured in darkness and then with a background light, and the shift in that potential during the period of light (light rise) is measured. The amplitude of the light rise is determined by the activity of ion channels on the basolateral membranes of RPE cells. Most *BEST1* mutations associated with Best’s disease are missense mutations [54], but another bestrophinopathy is late onset, referred to as adult-onset vitelliform macular dystrophy (AVMD; OMIM: 608161), and in addition to mutations in *BEST1* that lead to AVMD there are known mutations in three other genes associated with photoreceptors. Only AVMD patients with mutations in *BEST1* show a reduction during the EOG light rise test, indicating that normal bestrophin-1 functioning in RPE cells is critical for the light rise [57]. It is not known if *MITF* mutations affect the ERG light peak or EOG light rise response in humans. Other indicators of RPE cell’s physiological functioning are unknown except the ERG c-wave, a wavelet mainly mediated by RPE activity in some *Mitf* mouse mutations [17,58], but four known mutations in the *Mitf* gene in mice have been found to affect the BEST1-promoter, even those affecting different domains of *MITF*, related to the severity of the phenotype [46]. Less, in fact nearly nothing, is known about the interaction between *MITF* in the RPE and other ion channels expressed on these cells that are involved in ion transport or other physiological functions of the epithelium.

## 3. *Mitf* and the Vasculature of the Eye

In the postnatal eye, mouse *Mitf* is expressed exclusively in the RPE, even though it plays a complex role in transdifferentiating the RPE and neuroretina during development [8,15]. However, it is known that the RPE plays a role in retinal and choroidal vascular development and homeostasis through the release of various growth factors [59,60,61,62]. Some of these are pro-angiogenic, while others are anti-angiogenic. These growth factors include the angiogenic vascular endothelial growth factor (VEGF), the fibroblast growth factor (FGF), the platelet-derived growth factor (PDGF), and the anti-angiogenic pigment epithelium-derived factor (PEDF). It is clear now that, through the release of these factors, the RPE plays a role in retinal and choroidal vascular development and homeostasis [63] and cell migration during development [64], and that *MITF* interacts with all these factors in a complex manner.

VEGF is expressed in retinal Muller cells and during eye development in retinal astrocytes. The only source of the VEGF at the back of the eye is the RPE; isoforms VEGF120 and VEGF164 are primarily expressed there, with almost no detectable levels of VEGF188 [65]. It has been reported that neither the depletion nor knockdown of *MITF* expression affected VEGF expression in human RPE cells (ARPE-19). However, a reduction in Tfe3 expression in ARPE-19 cells reduces VEGF expression in these cells [66], suggesting that members of the MITF-Tfe family are involved in regulating VEGF expression in the RPE, but *MITF* is probably not involved. It has been established that for normal choriocapillaris development in mice, and indeed normal eye size and visual function, the expression of VEGF in the RPE is essential [67]. Other pigmented cells in the eye, particularly the melanocytes in the choroid, appear to play a role in these processes.

**Figure 1:**
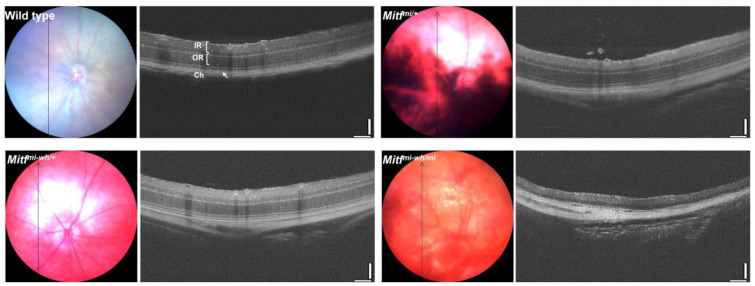
Morphological retinal alterations in mouse models of *Mitf* at 3 months of age. Representative optical coherence tomography (OCT) images were obtained from anesthetized mice with different mutations in the *Mitf* gene, showing mild to severe effects. The OCT scans were obtained from the right eye in all animals. Interestingly, the choroid (Ch) is progressively thick in all mutants. Retinal degeneration is evident in the *Mitf^mi-wh/mi^* mouse. RPE hypopigmentation is evident in all mutants. Changes in the retinal vasculature are present to some extent in all the mutants at 3 months of age, with the most dramatic effects being observed in the *Mitf^mi-wh/mi^* mouse. RPE (white arrow); IR, inner retina; OR outer retina; Ch, choroid.

Microphthalmia black-eyed-white mutant mice (*Mitf^mi-bw^*), which possess a spontaneous *Mitf* mutation involving the insertion of an L1 element into an intron [18] which abolishes the expression of the *Mitf-M* isoform, have a white coat but black eyes because they lack melanocytes while the RPE is normally pigmented and developed (Table 1). These mice have a much thinner choroid than wild type mice, and far less developed vascular layers in the choroid, while the retina appears normal in histologic sections [68]. Thus, it may be that there is an interplay between RPE cells and choroidal melanocytes in developing and maintaining the choroid and its vasculature, and that *MITF* may play a key role in establishing and regulating that interplay, but further work is needed to elucidate the processes involved. However, there is further evidence that supports such a role for *MITF*. As shown in Figure 1, through optical coherence tomography (OCT) scans of the eyes of mutant and wild type mice, we can see that, in several mouse mutants with mutations in the *Mitf* gene, there are changes in the thickness of the choroid (Figure 1). The animals received an intraperitoneal injection (IP) of anesthesia, consisting of ketamine (40 mg/kg) and xylazine (4 mg/kg), before OCT scanning. The eyes were treated with Mydriacyl (1% tropicamide, Alcon laboratories) drops to widen the pupils and Alcaine (proxymetacaine, Alcon laboratories) drops for corneal anesthesia. The eyes were kept moist by applying a thin layer of Methocel gel (2% methylcellulose, OmniVision, Santa Clara, CA, USA). Fundus images and retinal morphology were acquired from the animals’ eyes using a Micron IV SD-OCT system with a fundus camera. Photographs were obtained after visualizing the fundus, ensuring that the optic nerve was as centered as possible.

The OCT scans provide anatomical cross-sections of the retina, RPE, and choroid in living subjects, humans, or rodents, and allow for the measurement of the thickness of these structures. Additional studies are needed to elucidate the pathophysiological mechanism of the increased choroidal thickness in our mouse models of *Mitf*. In addition, histological sections of the eyes of *Mitf^mi-enu22(398)^/Mitf^mi-enu22(398)^* mutant mice show that choroidal melanocytes adjacent to the Bruch’s membrane are absent [16], as seen in *Mitf^mi-bw^* mice [68]. The *Mitf^mi-enu22(398)^/Mitf^mi-enu22(398)^* mutant mice have a pigmented and apparently normal RPE, and the retinal layers and ERG responses are normal as well [16]. The two mutants are strikingly similar with respect to eye phenotype, while the coat color differs, but the choroidal vasculature in *Mitf^mi-enu22(398)^/Mitf^mi-enu22(398)^* mutant mice has not been examined.

Another growth factor released by the RPE, but anti-angiogenic, is PEDF. This trophic factor is secreted primarily from the apical side of the RPE [69]. In addition to the RPE, it is expressed in photoreceptors, inner nuclear layer cells, and ganglion cells [70]. PEDF-deficient (PEDF^−/−^) mice show increased retinal vascular density compared to wild type mice, greater vessel obliteration during oxygen-induced ischemic retinopathy, and neovascularization, while the retinal cell layers and ERG responses to light are normal [71]. In human ARPE-19 cells, *MITF* upregulates the expression of *PEDF* in these cells, indicating that *PEDF* is a target gene of *MITF* [64]. Mice with a null mutation in the *Mitf* (*Mitf^−/−^*) show a decrease in PEDF protein levels and downregulation of the *Pedf* gene in the RPE and interphotoreceptor matrix. These *Mitf* mutant mice show progressive retinal degeneration, which can be partly restored by eye drops containing PEDF [72], although their retinal and choroidal vasculature has not been examined in detail. Given that the genes regulating the expression of two vital trophic factors, VEGF and PEDF in the RPE and choroid, involved in regulating retinal and choroidal vascularization, are *Mitf* target genes, it may be expected that *Mitf* mutant mice show some alterations in their retinal and choroidal vasculatures. However, aside from the three-dimensional structural analysis of choroidal blood vessels in *Mitf^mi-bw^* mice [68], very few analyses of the retinal vasculature in *Mitf* mutant mice have been conducted so far [73,74]. We found that *Mitf^mi-enu22(398)^/Mitf^mi-enu22(398)^* animals show an increase in the vascularization of the retina while *Mitf^mi-vga9/+^* animals show a decrease in the vascularization on the retina compared with control mice [74]. The retinal and choroidal vasculature of other *Mitf* mutant mice has not been examined in any detail, but the data so far indicate that other mutations in the gene should be able to shed further light on the role of *Mitf* in retinal vasculature.

**Table 1:** *Mitf* mutations discussed in this review with a strong phenotype in homozygous condition.

Genotype	Phenotype	Source
*Mitf^mi^*	White coat; eyes small and red; osteopetrosis; inner defects; incisors fail to erupt; deficiency of mast cells.	[7]
*Mitf^mi-rw^*	Colored marks around the neck; eyes small and red.	[75]
*Mitf^mi-bw^*	White coat with colored spots on rump and head; eyes small and red.	[76]
*Mitf^Mi-wh^*	White coat; eyes slightly pigmented and small.	[19]
*Mitf^mi-vga9^*	White coat; eyes small and red.	[7]
*Mitf^mi-vit^*	Initial markings on the thorax and abdomen; gradual loss of pigmentation in coat and eye; defective RPE–photoreceptor interactions.	[77,78,79]
*Mitf^Mi-or^*	White coat; eyes small and red; osteopetrosis; incisors fail to erupt.	[80,81]
*Mitf^Mi-H^*	White coat; eyelids are closed at birth.	[82]
*Mitf^Mi-b^*	White coat; reduced eye pigmentation.	[83]

## 4. *Mitf* Mutations and Microphthalmia

The first mutation induced in the mouse *Mitf* gene, by radiation [1], with a 3-bp deletion in the basic domain [7], produced one of the most severe phenotypes, which included microphthalmia, but only some mutations in the gene lead to microphthalmia, and its severity varies. Further examination of the development of the embryonic and postnatal eyes of *Mitf^mi/mi^* mice revealed that early neural retinal differentiation is not affected by the mutation (Table 1). The RPE in these mice has no pigmentation; at postnatal day 2, there are well-defined ganglion cell and inner plexiform layers in the retina, and both amacrine and horizontal cells are labeled by markers [14]. At 32 weeks postnatal, the outer nuclear layer (ONL) is completely absent, and the remaining retinal layers are either missing or greatly disorganized [14]. Both RPE differentiation and rod outer segments are affected in *Mitf^mi/mi^* mice, so clearly the loss of MITF function affects outer segment development. This is of interest because the formation of outer segments involves two stages: initiation and elongation. The RPE in *Mitf^mi/mi^* mice fails to develop microvilli on the apical side. The RPE basolateral surface is loosely organized but retains ezrin labeling. There may be a link between the failure of the RPE apical domain to form and the lack of rod outer-segment elongation [84], even though these photoreceptors express opsin, PDE, and peripherin. It appears that outer-segment initiation takes place in *Mitf^mi/mi^* mice, but elongation is not achieved [84].

**Figure 2:**
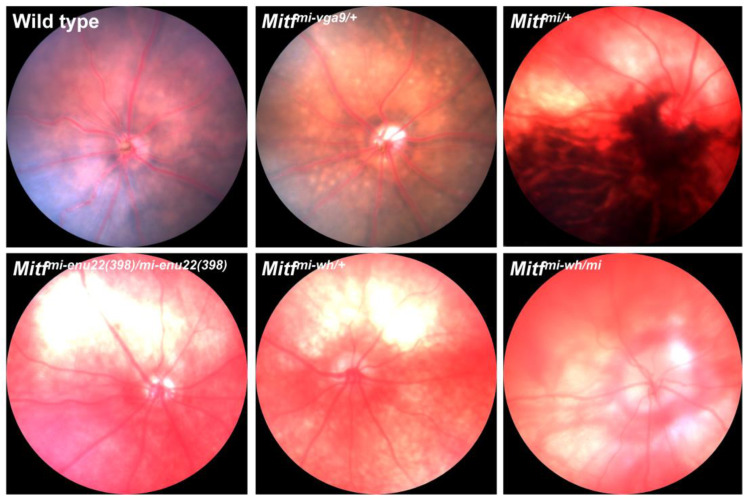
Representative fundus photographs of our mouse models of *Mitf* at 3 months of age showing mild to severe effects. The images were obtained from the right eye in all animals. Progressive loss of pigmentation, varying in degree, is evident in the mutants. The fundus images from *Mitf* mutants represent an allelic series of the gene, showing mild to severe effects. Adapted and modified with permission from García-Llorca et al. [16,17].

One of the most interesting mutations in the *Mitf* gene that leads to microphthalmia, and the second mutation discovered at the locus (and also induced by radiation), is the *Microphthalmia White* (*Mitf^Mi-Wh^*) mutation [2,85,86]. About half of the *Mitf* alleles in mice are recessive, so they only produce a phenotype in homozygous animals, but there are also dominant negatively acting *Mitf* alleles, which are genetically semi-dominant in mice, and the *Mitf^Mi-wh^* allele is one of these [76] (Table 1). The consequence of its being semi-dominant is that mice with heterozygous alleles have a fairly mild phenotype, while homozygotes have a more severe phenotype, with their coat being fully devoid of pigment [2]. However, aside from coat color, the eye phenotype in the homozygous condition is relatively mild, with the microphthalmia being far less severe than in the *Mitf^mi/mi^* mice [76,87]. The dominant negatively acting allele *Mitf^Mi-wh^* has an unusual phenotype due to a mutation of Ile212Asn in the DNA-binding domain; this allele shows interallelic complementation with respect to eye phenotype [76]. Interallelic complementation means that, in the case of a compound heterozygote comprising a combination of two *Mitf* mutants, its phenotype is less severe than the phenotype of either homozygote [18,76] (Table 1). The *Mitf^Mi-wh^* mutation is the only mutation at the *Mitf* locus to show interallelic complementation [87]. When the *Mitf^Mi-wh^* mutation is crossed with the severe *Mitf^mi^* mutation, the resulting *Mitf^Mi-wh/Mi^* compound heterozygote has a normal eye size, although pigmentation is still lacking in the eyes and coat [86]. Some, but not all, other *Mitf* mutations are complemented when combined with the *Mitf^Mi-wh^* mutation [76]. The molecular lesion of the *Mitf^Mi-wh^* mutation is that isoleucine at position 212 is changed to asparagine at the middle of the DNA-binding basic region of the protein, and it has been shown that DNA binding is affected by the lesion and that this is dependent on splice variants [4,76]. Although the microphthalmia in the *Mitf^Mi-wh^* homozygous mutant mice is mild, and the eyes are slightly pigmented, the RPE is without pigmentation, there is severe retinal degeneration, and the corneal ERG shows no response to light stimuli at 16 weeks of age [58]. Heterozygous *Mitf^Mi-Wh/+^* mice have eyes of normal size that are dark ruby in color, but a gray, diluted coat color. Bright field fundus images obtained from *Mitf^Mi-Wh/+^* mutant mice show hypopigmentation of the fundus, with large non-pigmented areas but no pigment mottling (Figure 2).

Another *Mitf* mutation induced by irradiation, in this case gamma irradiation, leading to microphthalmia is the Oak Ridge mutation, or *Mitf^Mi-or^*, which was induced at the Oak Ridge National Laboratory in Oak Ridge, Tennessee [80,81] (Table 1). The most extreme eye defects are seen in *Mitf* mutations in the basic region of the bHLH-Zip domain, including the Oak Ridge mutation. Homozygotes have small, red, or absent eyes, and a white coat color, while heterozygotes show a slight dilution of coat color. Compound heterozygotes with *Mitf^Mi-Wh^/Mitf^Mi-or^* have a normal eye size but reduced eye pigmentation, and a white coat color [76]. Little is known about the eye phenotype and the conditions of the retina and RPE in heterozygotes with the Oak Ridge mutation.

In addition to spontaneous mutations found in the mouse *Mitf* gene, and those induced by irradiation that leads to microphthalmia, mutations have been found that were induced by chemical mutagens, and then first detected via examination of the phenotype. One of the most effective and efficient chemical mutagens is *N*-ethyl-*N*-nitrosourea (ENU), which primarily induces point mutations [88]. At the MRC Harwell in Oxfordshire, England, two ENU-induced point mutations in the *Mitf* gene were found, one of which showed anophthalmia in the homozygous condition and was named *Microphthalmia Harwell* (*Mitf^Mi-H^*) [82] (Table 1). The homozygotes were completely unpigmented, and the eyelids were closed at birth. Histology showed a small amount of eye tissue in the orbit, but this was not comparable to intact or misshapen eyes. Heterozygotes have pale patches on their coat, belly, and head, and pale ears, feet, and tails. An eye examination of heterozygotes showed mild iris transillumination, but no other eye abnormality was found (based on bio microscopy and ophthalmoscopy) [82]. It is not clear whether heterozygotes have altered retinal or RPE function or changes in the pigmentation of the RPE and choroid. Two other ENU-induced semidominant mutations in the *Mitf* gene have been described, which have somewhat different phenotypes than *Mitf^mi^^/mi^* and *Mitf^Mi-or^* mice, such as the absence of osteopetrosis, but the homozygotes still have severe microphthalmia and are unpigmented, while heterozygotes, in some cases, have pigmented patches in the coat and a normal eye size. These are the *Mitf^mi-enu5^* and *Mitf^mi-bcc2^* mutations [89]. The mutations, in both cases, affect the DNA-binding domain. The former mutation severely affects hearing in both homozygotes and heterozygotes, while hearing is normal in *Mitf^mi-bcc2^* mice. Mating experiments show that the *Mitf^mi-enu5^* and *Mitf^mi-bcc2^* mutations do not complement each other [89]. Retinal and RPE function, fundus appearance, and other aspects of the eye phenotype have not been examined in detail in *Mitf^mi-enu5^* and *Mitf^mi-bcc2^* heterozygotes. Finally, a *Mitf* mutation that leads to microphthalmia was induced by transgenic insertion and 882 bp deletion [3]. Several transgenic lines were established, and one of them, *VGA-9*, had an easily visible phenotype with transgene homozygosity, showing microphthalmia and loss of pigmentation, in addition to cochlear abnormalities and hearing defects [3]. The transgenic line was originally established to examine the promoter regions of the arginine vasopressin (Avp) and oxytocin genes [9,90]. That failed, but instead white mice appeared as an offspring of the Avp line, i.e., the ninth mouse examined after injection with a vasopressin-β-galactosidase transgene, and these were given the name VGA-9 [9]. The *Mitf^mi^^-vga9^* mutation is a loss-of-function mutation, with greatly reduced expression of the gene [7], and thus microphthalmia is severe in homozygotes and their coat is completely white [76] (Table 1). We examined the eye phenotype of *Mitf^mi^^-vga9/+^* mice in detail at the age of 3 months and found that they have a normal eye size and overall eye pigmentation appears normal, while bright fundus images reveal a minor hypopigmentation with discrete yellow lesions of the fundi and pigment mottling spots scattered throughout the fundus (Figure 2). Both rod and cone ERG responses are normal, and the thickness of the retina is not different from that of control animals [16]. The retinal vasculature of *Mitf^mi^^-vga9/+^* mice is comparable to that of wild type mice [16], except that the combined retinal venular diameter is significantly larger in the mutants, suggesting subtle effects of the mutation on the vasculature [74] but with limited consequences for vision.

## 5. *Mitf* and Postnatal Retinal Degeneration

One of the earliest genotypes with mutations in the microphthalmia gene examined with respect to eye phenotype was the Vitiligo (C57BL/6-mi^vit^/mi^vit^) mouse [78,79,91] (Table 1). This was first reported as a potential model for the dermatologic disease vitiligo [77], but later it was found that pigmentation in the eye was reduced as well [92], similar to what occurs in human vitiligo patients. The molecular lesion that is responsible is an amino acid substitution in the first helix [19], and the mutation is recessive and spontaneous [19,76]. Abnormalities in the RPE are known to be evident in homozygous *mi^vit^/mi^vit^* mice before changes in photoreceptor morphology appear [93]; published fundus images suggest depigmented areas and star patterns [94]. Fewer phagosomes are present in the RPE of *mi^vit^/mi^vit^* mice than in wild type mice, but it is not clear if that is a primary defect or a consequence of a gradual reduction with time in the photoreceptor outer segments [95,96]. RPE microvilli are short or absent in the RPE cells of *mi^vit^/mi^vit^* mice; the cells show abnormal basal infoldings and the accumulation of ROS debris, which suggests that the RPE–photoreceptor interaction is defective [96]. In the retina, both electrophysiological and histopathological findings indicate that there is a gradual degeneration of rod photoreceptors in homozygous but not heterozygous mutants, starting at 3 weeks post-partum [79], and a delay in the implicit times of the dark-adapted a- and b-waves [91]. Therefore, it is unclear if cones and RPE function, as assessed by the ERG c-wave, are affected in these mice.

A semi-dominant spontaneous mutation that leads to postnatal retinal degeneration is the *Microphthalmia-brownish* (*Mitf^Mi-b^*) mutation. Both homozygous and heterozygous mutants have a normal eye size, and normal bone formation, but homozygotes have reduced eye pigmentation, a completely white coat, and pale ears and tail [83,97]. The coat color of heterozygotes is diluted, with a “brownish” cast, and eye pigmentation appears diluted [18,83]. The mutation is a point mutation in helix 2 of the HLH domain, with a lesion at G244E, and it does not have an effect on the expression of the gene or dimerization but has an effect on the ability of the mutant protein to bind DNA [83]. Since the mutation is semi-dominant, its effect on coat color and pigmentation in general is strikingly different between homozygotes and heterozygotes. The mutation affects the pigmentation of the RPE and choroid of the eye, with no pigmentation of these structures in homozygotes, while there is normal pigmentation of the RPE in heterozygotes but only a few foci of pigmentation in the choroid [83]. No bright field fundus images from *Mitf^Mi-b^* have been presented; however, given the reduced pigmentation of the choroid, it is likely that such images would reveal hypopigmentation [16]. From these and other aspects of the phenotype, it appears that the *Mitf^Mi-b^* mutation primarily affects melanocyte survival but not necessarily function [83]. It is not known if there is retinal degeneration or altered retinal function in heterozygous mutants (*Mitf^Mi-b^/^+^*). It is also not clear why retinal degeneration occurs in the homozygous mutants; it may be that the mutation affects other RPE functions than pigmentation and melanocytes.

The original *Mitf^mi/mi^* mutation, first induced in the *Mitf* gene by radiation, produced severe microphthalmia [1,2], as described above, and was semidominant [4]. However, *Mitf^mi/+^* mice have a normal eye size and, in most cases, a normal coat color, although some rare cases show small white spots on the belly, head, or tail [2]. We recently examined the eye phenotype of *Mitf^mi/+^* mice and found that progressive cone–rod retinal dystrophy and RPE dysfunction occurs over about 18 months, and progressive changes in the pigmentation of the fundi occur over the same period [17]. The c-wave of the ERG, which primarily indicates RPE function, is significantly reduced in *Mitf^mi/+^* mice at 1 month, earlier than ERG components related to neural activity (a- and b-waves), suggesting that defects in RPE function precede retinal dystrophy. The fundi at 3 months of age show large non-pigmented lesions in the superior half of the fundus, while there are hyper-pigmented lesions in the inferior half (Figure 2), but the hyper-pigmentation is reduced by age, and fundus images for ages up to 18 months indicate progressive, age-related hypopigmentation of their fundi. In addition, histologic examinations show a gradual thinning of all retinal layers [17]. However, compound heterozygotes with the *Mitf^mi^* mutation and *Mitf^Mi-Wh^* mutation crossed (*Mitf^Mi-Wh^*/*Mitf^mi^*) provide an interesting example of the interallelic complementation of these mutations [86]. In each of these mutations, in homozygous conditions, there is severe or intermediate (in the *Mitf^Mi-Wh^* mutation) microphthalmia, as described above, but in the *Mitf^Mi-Wh^*/*Mitf^mi^* compound, heterozygotes’ eye size is normal while pigmentation is still lacking in the coat and eyes [86]. Combining the two alleles in the same gene complements them; this is referred to as interallelic or intergenic complementation [76,87]. The fundi in these mice at 3 months of age have a widespread lack of pigmentation and lesions in the RPE that appear large (Figure 2), but no pigment mottling. The rod- and cone-driven ERG responses are completely flat at 3 months of age, and a histological analysis of the retinal layers at that age shows that the photoreceptor layer and the IPL are absent from their retinae [16]. Thus, interallelic complementation may induce the development of the full eye size but may not prevent severe and early retinal degeneration. That is further supported by findings with respect to the eye in another compound heterozygote, *Mitf^Mi-Wh^*/*Mitf^mi-sp^* mice.

The *Mitf^mi-sp^* mutation was originally found among five mice in a colony of *Mitf^Mi-Wh^* mice crossed with C57BL/6 mice, and occurred in a C57BL/6 parent [98,99]. Mice homozygous or heterozygous for *Mitf^mi-sp^* are indistinguishable from their wild type littermates with respect to coat color, eye size, and pigmentation [76,99], although tyrosine activity in the skin is reduced in both alleles compared to wild type mice [19,99], probably due to a reduction in the total number of skin melanocytes. The mutation involves a cytosine insertion in splice acceptor 2, thus affecting splicing, which, in turn, results in a protein lacking the six amino acid insert sequence; the gene is missing an 18 bp alternatively spliced exon [19,97]. An examination of retinal structure and function in the eyes of mice homozygous for *Mitf^mi-sp^* showed that both rod- and cone-driven ERG responses are normal, including the c-wave, indicating that RPE function is normal, and histologic sections showed that all retinal layers are intact, with pigmentation in the RPE and choroid comparable to that of wild type mice [58]. It was found that *Mitf^Mi-Wh^*/*Mitf^mi-sp^* compound heterozygotes have a tan coat color with white spots on the coat, but normal eye size [76,98,99], with a phenotype which is intermediate between the two homozygotes, and thus lacks any apparent complementation [76]. ERG recordings obtained at 16 weeks of age showed that rod- and cone-driven responses were reduced, and the c-wave correspondingly reduced, indicating a rod–cone dystrophy in their retinae. The data indicate that the defect is primarily photoreceptor degeneration at that age, but it is not clear if the dystrophy is progressive. Histologic examination of the eyes of *Mitf^Mi-Wh^*/*Mitf^mi-sp^* mice at 16 weeks of age shows a selective thinning of the photoreceptor and RPE layers, and a reduction in RPE pigmentation, although fundus images are not available [58]. Thus, both semi-dominant and recessive mutations at the mouse *Mitf* locus can lead to retinal degeneration in normal-size eyes, and this appears to apply to both those that are spontaneous and those induced by radiation or chemical mutagens.

In humans, *MITF* is implicated in the development of WS2, Tietz syndrome, and COMMAD syndrome. WS2 is a genetic condition inherited dominantly, causing hearing impairments and reduced hypopigmentation in the eyes, skin, and hair. WS2 is primarily caused by an anomaly in the development of melanocytes and the expression of key genes involved in melanin production, such as *TYR*, *TYRP1*, and *TYRP2,* is altered [6,100]. in vitro studies have revealed a correlation between WS2 and *MITF* mutations, particularly the c.650G>T (p.R217I) and c.575delC (p.T192fsX18) mutations, found in approximately 15% of cases. Only the R2171 *MITF* function is diminished, and the T192fsX18 *MITF* variant cannot activate the *TYR*-promoter [101]. In addition, another study has shown that WS2 is linked to a genetic alteration at a termination codon [102]. Tietz syndrome, like WS2, is genetically dominant but much less prevalent, with symptoms that are more severe, including profound deafness from birth, continuous hypopigmentation, vivid blue irises, and a lack of RPE pigmentation [23]. The combination of coloboma, osteopetrosis, microphthalmia, macrocephaly, albinism, and deafness are known as COMMAD. It is associated with a biallelic *MITF* mutation, leading to issues in optic-fissure closure and bone development [24].

## 6. Conclusions

The *Mitf* transcription factor plays a key role in eye development, but there is increasing evidence indicating that it plays a wider role in the formation of eye structure and the functioning at the back of the postnatal eye. The *Mitf* gene is abundantly expressed in neural-crest-derived melanocytes. However, its expression in the RPE is strong and it is clearly an important source of *Mitf* in the eye. *Mitf* mutations have dramatic effects on eye size in some cases, on the pigmentation of the RPE and choroid, and, in some cases, on the retina. The MITF gene’s protein is vital for the development and function of various cell types, including osteoclasts, melanocytes, and the RPE. Most known *Mitf* alleles have been examined with regards to melanocytes, RPE cells, and osteoclasts only, while their effect in other cells have been examined much lesser extent. It is known that the expression of some of the ion channels on the RPE is affected by MITF. However, the exact role of MITF in RPE ion transport is still unknown. Similarly, there are data indicating that MITF in the RPE may affect the retinal and choroidal vasculatures, via the regulation of growth factor release from the RPE, but the available data relevant to that question are limited. The integrity and function of both photoreceptors and RPE rely on MITF control and the regulation of numerous essential functions within the RPE, as demonstrated by the effects of *Mitf* mutations in mice on the retinal and RPE structure and function. Exploring the role of MITF in pathways associated with retinal diseases is crucial due to its impact on a wide array of biological functions in RPE cells, offering potential for therapeutic advancements.

## Data Availability

No new data were created or analyzed in this study.
